# Fluorine and chlorine substituted adamantyl-urea as molecular tools for inhibition of human soluble epoxide hydrolase with picomolar efficacy

**DOI:** 10.1080/14756366.2023.2274797

**Published:** 2023-11-17

**Authors:** Vladimir V. Burmistrov, Christophe Morisseau, Dmitry V. Danilov, Boris P. Gladkikh, Vladimir S. D’yachenko, Nikolay A. Zefirov, Olga N. Zefirova, Gennady M. Butov, Bruce D. Hammock

**Affiliations:** a Volgograd State Technical University, Volgograd, Russia; b Department of Entomology and Nematology, and Comprehensive Cancer Center, University of California, Davis, CA, USA; c Department of Chemistry, Technology and Equipment of Chemical Industry, Volzhsky Polytechnic Institute (branch) Volgograd State Technical University, Volzhsky, Russia; d Department of Chemistry, M. V. Lomonosov Moscow State University, Moscow, Russia

**Keywords:** Soluble epoxide hydrolase inhibitors, adamantane, urea, diurea, chlorine substituted adamantanes, fluorine substituted adamantanes

## Abstract

Series of 1,3-disubstituted ureas and diadamantyl disubstituted diureas with fluorinated and chlorinated adamantane residues were shown to inhibit human soluble epoxide hydrolase (sEH) with inhibition potency ranging from 40 pM to 9.2 nM. The measured IC_50_ values for some molecules were below the accuracy limit of the existing *in vitro* assays. Such an increase in activity was achieved by minimal structural modifications to the molecules of known inhibitors, including 4-[*trans*-4-(1-adamantylcarbamoylamino)cyclohexyl]oxybenzoic acid. For the chlorinated homologue of the latter the sharp jump in inhibitory activity can be (according to molecular dynamics data) the result of interactions – Cl-π interaction. Considering the extremely high inhibitory activity, acceptable solubility and partial blockage of metabolically sensitive centres in their structures, some compounds are of interest for further *in vivo* biotesting.

## Introduction

A wide range of mammalian biochemical pathways are regulated by enzyme soluble epoxide hydrolase (sEH), which is involved in the metabolism of epoxyeicosatrienoic acids (EETs) to the corresponding diols.^1^ Experimental manipulations of sEH are frequently achieved by its inhibition. It has been shown that such inhibition leads to improved microcirculation and promotes tissue repair after myocardial infarction and ischaemic stroke. It also reduces systemic arterial pressure, increases diuresis, and exerts anti-inflammatory, neurogenic and antidepressant-like effects.^2–9^

Most of the potent sEH inhibitors published to date contain urea moiety, which mimics epoxide transition state in the active site of the enzyme.^10^ Among the highly active inhibitors are 1,3-disubstituted ureas containing an adamantane residue, for example, 1-(1-acetyl-piperidin-4-yl)-3-adamantan-1-yl-urea (APAU)^11^ (4-[*trans*-4–(1-adamantylcarbamoylamino)cyclohexyl]oxybenzoic acid (*t*-AUCB, **1a**)^12^ and its homo- or chlorinated derivatives (**1b**, **1c** respectively)^13^ or diadamantyl disubstituted diureas of general formula **2.**^13^^,^^14^ Some of them have inhibitory activity *in vitro* in the low nanomolar concentration range (IC_50_: 0.5–2 nМ).

Despite the high activity of adamantane-containing sEH inhibitors, their use as molecular tools for *in vivo* studies is limited, since they often have poor pharmacokinetic properties. The latter include low metabolic stability caused by the high lipophilicity of adamantane, which is prone to oxidation by metabolic enzymes.^15^^,^^16^ Different attempts were undertaken to solve the problem: incorporation of hydrophilic groups into the adamantane core^13^^,^^17^ or replacement of the latter with more polar groups (see, e.g. ref. 18) etc. These attempts met with limited success, since adamantane fits the hydrophobic pocket in the binding site of sEH, and the insertion of polar groups often leads to a decrease in activity. Recently, however, it has been shown that the replacement of the adamantane residue in *t*-AUCB with more lipophilic fluorinated and chlorinated benzohomoadamantane leads to compounds **3а** and **3b** with good metabolic stability and water solubility.^19^ Perhaps this is due to the partial blocking of metabolically sensitive centres by halogen atoms, which is widely used in drug design^20–22^ Previously, we synthesised series of fluorinated and chlorinated analogs of *t*-AUCB derivative **1b** and diureas **2**,^13^^,^^14^ namely compounds **9**, **10**, **11a–i**, **12a–g** and **12i.**^23^^,^^24^ Given the remarkable microsomal stability and inhibitory efficiency of compounds **3а**,**b** we proposed to carry out the primary bioscreening of the series **9**, **10**, **11a–i**, **12a–g** and **12i**. In this communication, we present results of biotesting, which showed an unexpectedly sharp increase in inhibitory activity of some compounds to picomolar concentration range. Possible explanations of these results by molecular modelling methods and the data on the water solubility of the tested compounds are also presented.

## Results and discussion

Series of 1,3-disubstituted ureas (**9, 10**) and diadamantyl disubstituted diureas with fluorinated and chlorinated adamantane residues (**11a–i**, **12a–g** and **12i**) was obtained previously by a multi-step procedure with a key stage being the reaction of 1-fluoro-3-(isocyanatomethyl)adamantane and 1-chloro-3-(isocyanatomethyl)adamantane with *trans*-4-((4-aminocyclohexyl)oxy)benzoic acid or the corresponding diamines.^23^^,^^24^ Some physicochemical characteristics of the compounds are presented in Tables 1 and 2.

**Table 1.** Human sEH inhibition data and some physicochemical characteristics of ***t*-AUCB** analogs **9** and **10**.

	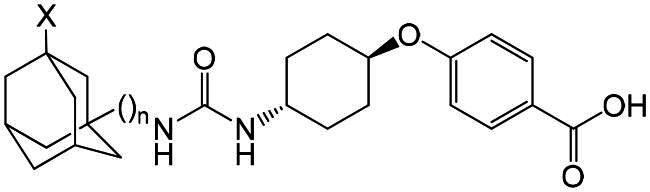						
Cmpd	X	*n*	LogP_calc_^a^	M.p., °C	Saq_calc_^a^, μM	Saq^b^, μM	IC_50_^c^, nM
**9**	F	1	4.01	85–86	10.3	150 ± 20	0.6
**10**	Cl	1	4.02	192–193	5.78	160 ± 20	0.04
***t*-AUCB**	H	0	3.70		10.9	160 ± 20	2
***t*-TUCB**	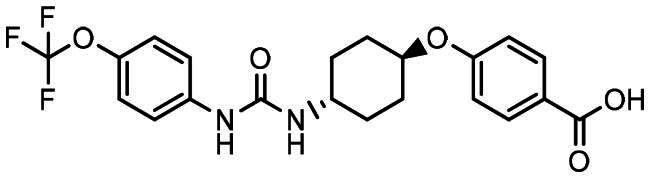		–	–	–	–	0.9

^a^Logarithm of the octanol–water partition coefficient (mean value) and aqueous solubility (ESol method^25^) calculated using Swiss ADME software.^26^

^b^Aqueous solubilities measured in sodium phosphate buffer (pH 7.4, 0.1 M) containing 1% of DMSO by turbidimetric assay.

^c^Half maximal inhibitory concentration determined as described in Ref. 29, means of three independent experiments. For structural formula of ***t*-AUCB** see introduction.

**Table 2.** Human sEH inhibition data and some physicochemical characteristics of compounds **11a–i**, **12a–g**, **12i**.

	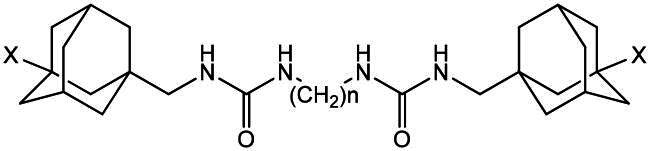						
Cmpd	X	n	LogP_calc_^a^	M.p., °C	Saq_calc_^a^, μM	Saq^b^, μM	IC_50_^c^, nM
**11a**	F	2	3.92	78–79	49.5	150 ± 20	9.2
**11b**	F	3	4.32	87–88	28.0	150 ± 20	1.8
**11c**	F	4	4.61	142–143	16.0	180 ± 20	0.5
**11d**	F	5	4.93	75–76	9.07	140 ± 20	0.4
**11e**	F	6	5.28	82–83	5.13	150 ± 20	0.04
**11f**	F	7	5.61	77–78	2.23	200 ± 20	0.04
**11g**	F	8	5.99	95–96	0.972	180 ± 20	0.04
**11h**	F	9	6.33	73–74	0.423	200 ± 20	0.05
**11i**	F	10	6.71	76–77	0.184	150 ± 20	0.05
**12a**	Cl	2	4.23	222–223	15.2	60 ± 10	5.1
**12b**	Cl	3	4.58	194–195	8.59	80 ± 10	0.04
**12c**	Cl	4	4.87	213–214	4.93	60 ± 10	0.04
**12d**	Cl	5	5.19	157–158	2.79	70 ± 10	0.04
**12e**	Cl	6	5.63	148–149	1.57	60 ± 10	0.04
**12f**	Cl	7	5.85	164–165	0.685	80 ± 10	0.04
**12g**	Cl	8	6.23	187–188	0.298	60 ± 10	0.1
**12i**	Cl	10	6.92	189–190	0.0566	60 ± 10	0.04
**DAMH**	H	6	–	–		65 ± 5	0.4

^a^Logarithm of the octanol–water partition coefficient (mean value) and aqueous solubility (ESol method^25^) calculated using Swiss ADME software.^26^

^b^Aqueous solubilities measured in sodium phosphate buffer (pH 7.4, 0.1 M) containing 1% of DMSO by turbidimetric assay.

^c^Half maximal inhibitory concentration determined as described in Ref. 29, means of three independent experiments. **DAMH** – 1,1'-(hexane-1,6-diyl)bis(3-(adamantan-1-ylmethyl)urea).^13^

Noteworthy is the irregularity in the change in the melting points of substances observed within each series. Disubstituted diureas containing fluorinated adamantane (**11**) generally have a much lower melting points than those with the chlorine substituent (**12**), while the melting points of the 1,3-disubstituted ureas **9** are **10** are reversed. The most obvious irregularity is a sharp jump in the melting point (confirmed in several independent measurements) for compound **11c**, which turns out to be approximately twice as high as that for the other substances of series **11**. These melting temperature aberrations obviously result from the influence of different structural factors (the length of the spacer between urea groups and the volume of the halogen substituent in adamantane) on a complex system of intra- and intermolecular interactions in the structures under consideration.

The water solubility of compounds measured in the turbidimetric assay is inversely related to their melting points in the series **11** and **12** (Table 2). The solubility of fluorine containing diureas **11** is twice as high (140–200 μM) as that of their chlorine containing analogues **12** (60–80 μM). Interestingly, the solubility of compounds **9** and **10** and *t*-AUCB is almost the same (Table 1) and chlorine containing diurea **12e** demonstrates equal solubility as its non-substituted analogue 1,1′-(hexane-1,6-diyl)bis(3-(adamantan-1-ylmethyl)urea) (DAMH, Table 2). Basically, no correlation was observed between the experimentally measured values of water solubility and those calculated by ESol method^25^ integrated to Swiss ADME software,^26^ as well as with the values of the logarithm of the octanol–water partition coefficient (logP) calculated in this software (see Tables 1 and 2). As expected, in series **11** and **12**, the calculated values systematically change depending on the number of methylene groups, while the measured values of water solubility within each series of diureas are practically independent on the length of the spacer between two urea groups. This trend is also linked to some features of intra- and intermolecular interactions of the considered structures in water.

The inhibitory potency of the compounds **9**, **10**, **11a–i**, **12a–g** and **12i** against the human sEH was studied using a kinetic fluorescent assay,^27^ *trans-*4-((4–(3-(4-(trifluoromethoxy)phenyl)ureido)cyclohexyl)oxy)benzoic acid (*t*-TUCB)^28^ and DAMH^13^ were used as positive controls. Both compounds were tested in our previous studies^13^^,^^28^ and had IC_50_ values 0.4 nM and 1 nM respectively in the earlier version of the assay with a measurement limit of 0.4 nM. In the present study we used a test with increased measurement accuracy up to 0.04 nM and got the same IC_50_ values for DAMH (Table 2) and for *t*-TUCB (Table 1).

The first interesting and unexpected result of the bioscreening was a sharp (by about an order of magnitude) increase in the inhibitory activity of chlorine containing compound **10** compared to its fluorinated (**9**) and unsubstituted analogues (**1b**). Thus, the activity of compound **10** is in the picomolar concentration range (Table 1). Theoretically, such a sharp increase in potency can be a consequence of both a decrease in the desolvation energy of the ligand with a bulky hydrophobic chlorine atom and the participation of the latter in additional interaction with the protein (hydrophobic, dipole–dipole, halogen, and, theoretically, even covalent bond etc.). To assume about the origin of the interactions we performed molecular dynamics simulations for the complex sEH − **10**.

**Figure 1.**
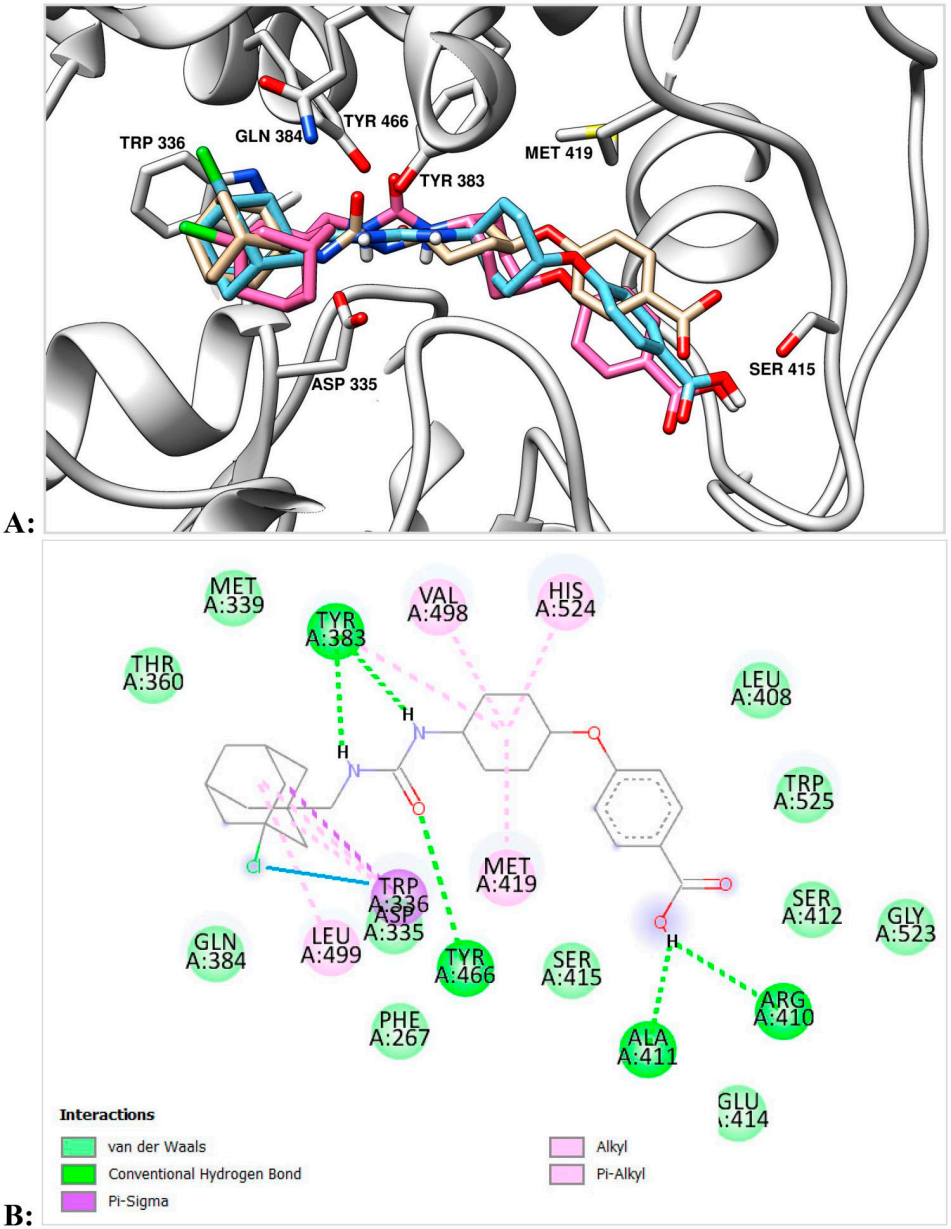
(A) Final view of the complex of **10** with sEH (PDB ID: 5AM3) predicted by molecular dynamics (in pink) and predicted by molecular docking (in light blue). Molecule of *t*-AUCB (**1a**) is shown in beige for comparison. The inhibitors are represented by the ball-and-stick model, selected amino acid residues are represented by coloured sticks, hydrogen atoms are omitted for clarity. Protein secondary structure is shown by ribbons. (B) A two-dimensional diagram of the interactions of compound **10** with sEH (Cl – π interaction is shown specially by a solid blue line).

Proposed binding modes of **10** with the sEH (PDB ID: 5AM3) obtained by molecular dynamics and molecular docking are presented at the Figure 1(A).

As can be seen from the Figure 1(A), urea moiety of **10** is located within the active site of enzyme and forms hydrogen bonds with Tyr383, Tyr466, and Asp335 residues. Adamantane moiety is exposed within the hydrophobic pocket formed by Trp336, Met 339, Ile363, Phe381, Leu499 residues. According to the docking data, the position of the adamantane ring in compound **10** is almost identical to that for *t*-AUCB, with the chlorine atom also being in the mentioned hydrophobic pocket. Simulation of molecular dynamics reveals, however, some changes in the position of the ligand from that predicted on the basis of molecular docking, namely, a slight compaction of the molecule and a deviation of the position of adamantane in depth (Figure 1(A,B)). In this case, the position of the chlorine atom is close to orthogonal with respect to the benzene ring of the Trp336 residue (at the distance 3.5 Å). This location is very close to the “face-on” Cl – π interaction, which, according to calculations, can provide an additional gain in binding energy of 2.01 kcal/mol (see in details in Ref. 29) Note that the fluorine substituent can theoretically also form a similar interaction, but it is more sensitive to the electronic characteristics of the aromatic fragment and occurs at a shorter distance.^30^

The presence of a methylene spacer between the adamantane and urea moieties gives molecule **10** some conformational flexibility compared to *t*-AUCB (**1a**) and seems to be important for the “face-on” Cl – π interaction (Figure 1). This conclusion is indirectly confirmed by the fact that the IC_50_ value for the chlorine derivative of *t*-AUCB, which does not contain the indicated methylene bridge (**1с**), is 1.4 nM,^13^ i.e. more than an order of magnitude higher than for compound **10**. Thus, according to molecular modelling data, the high inhibitory activity observed for compound **10** is the result of not a covalent or halogen bonding, but interactions – Cl-π interaction. This makes it interesting to further examine the sEH–**10** complex by X-ray crystallography.

**Figure 2.**
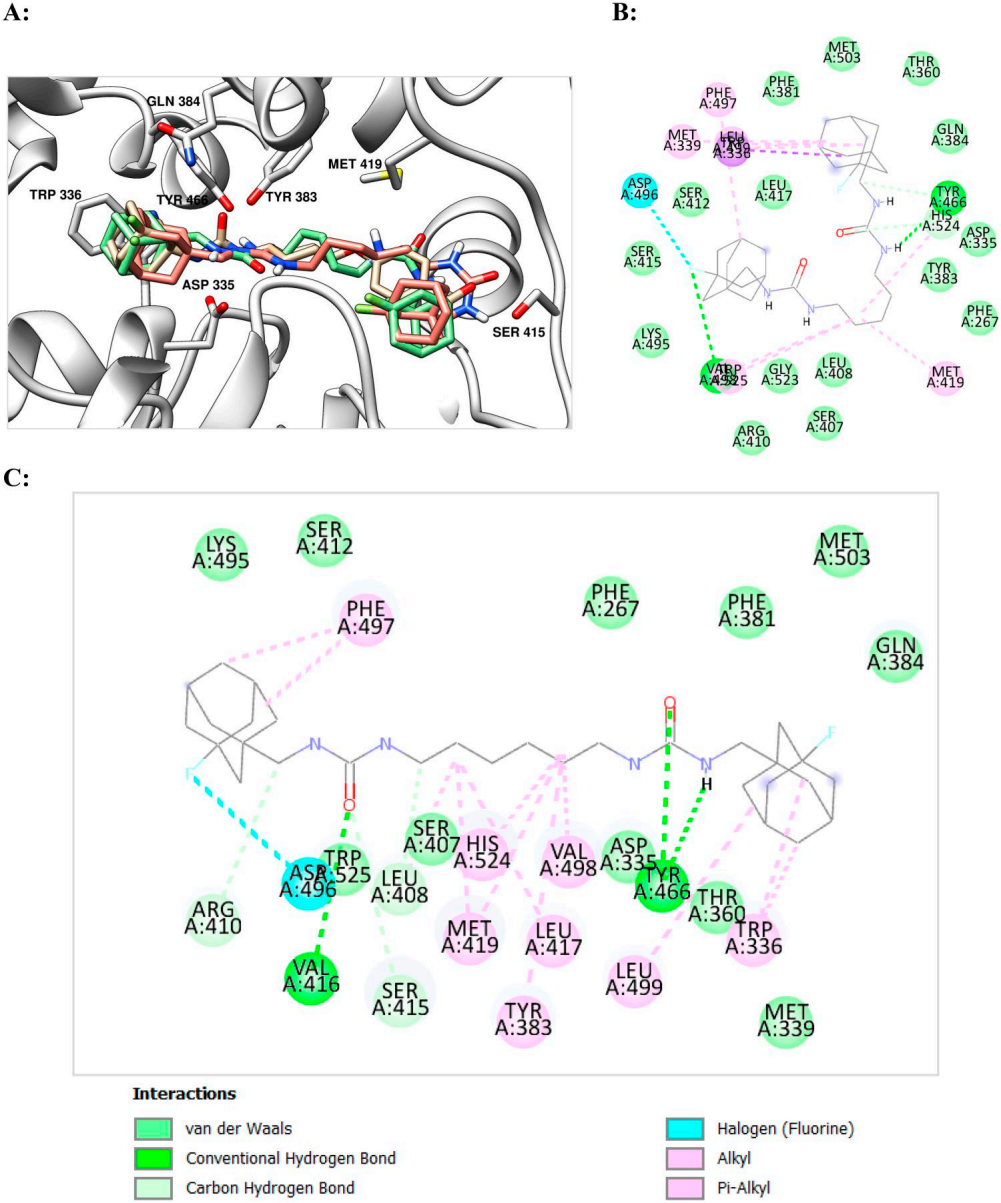
(А) Positions of compounds **11d** (in green) and **11e** (in salmon) with sEH predicted by molecular docking (in light blue). Molecule of *t*-AUCB (**1a**) is shown in beige for comparison. The inhibitors are represented by the ball-and-stick model, selected amino acid residues are represented by coloured sticks, hydrogen atoms are omitted for clarity. Protein secondary structure is shown by ribbons. (B,C) Two-dimensional diagrams of the interactions of compounds **11d** (B) and **11e** (C) with sEH. More essential interactions with residues of the active site for the second compound are clearly visible.

In the series **11** and **12** diureas with ethylene spacer between two urea groups (**11a** and **12a**) possess the lowest activity among the studied compounds (Table 2). This data is in accordance with the results of our previous study for the series of structurally similar unsubstituted diureas^18^ and is explained with the hydrophobic nature of the sEH active site. All other compounds of the series **11** and **12** were highly active, eleven diadamantyl disubstituted diureas with fluorinated and chlorinated adamantane residues demonstrated IC_50_ values as low as 0.04 nM. Chlorine containing compounds were more potent against sEH than their fluorine analogues, but the latter, starting with compound **11e** with six methylene spacer, also show picomolar activity. According to molecular modelling data, the difference in activities by an order of magnitude for compounds **11d** and **11e** can be explained by the possibility of the latter to form markedly more essential interactions with residues of the active site of the enzyme (see Figure 2(A–C)).

It should be emphasised, that it is hard to define the exact correlation of inhibitory activity with the length of methylene spacer between the urea groups in the series of chlorine derivatives **12**, because the measured IC_50_ values for some molecules were so low, that surpassed the accuracy limit of the assay. For example, for compound **12d** IC_50_ was 0.002 nM (2 pM), which was then reduced to the value of the measurement limit. Considering the extremely high inhibitory activity and acceptable solubility of the obtained substances it is of interest to further study their *in vivo* activity. The presence of partially blocked metabolically sensitive centres in their structures, combined with data on good metabolic stability for compounds **3a**,**b**,^19^ suggests that at least the metabolic stability of the ***t*-AUCB** derivative, compound **10**, may be better than that of the parent molecule.

## Conclusions

Series of 1,3-disubstituted ureas and diadamantyl disubstituted diureas with fluorinated and chlorinated adamantane residues were shown to inhibit human soluble epoxide hydrolase with inhibition potency ranging from 40 pM to 9.2 nM. The measured IC_50_ values for some molecules were below the accuracy limit of the existing *in vitro* assays. Such an increase in activity was achieved by minimal structural modifications to the molecules of known inhibitors, and for the chlorinated homologue of ***t*-AUCB**, compound **10**, it can be (according to molecular dynamics data) the result of interactions – Cl-π interaction. In general, some compounds of the presented series replenish the number of extremely potent inhibitors of human soluble epoxide hydrolase.

## Experimental

*The compounds* **9**, **10**, **11a–i**, **12a–g** and **12i** were obtained previously, following the method described in Refs. 23,24.

Logarithm of the octanol–water partition coefficient (Log P, mean value) was estimated using Swiss ADME software,^26^ aqueous solubility (Saq) was calculated by ESol method^25^ using Swiss ADME software.^26^

*Solubilities were measured in sodium phosphate buffer (pH 7.4, 0.1 M) containing 1% of DMSO by turbidimetric assay*.

Recombinant human and recombinant rat sEH were produced in insect High Five cells using recombinant baculovirus expression vectors, and purified by affinity chromatography as reported previously.^31^^,^^32^ Each enzyme appeared as a single band (0.3 μg loading) with an estimated purity of more than 95% by Coomassie Brilliant Blue staining following SDS-PAGE separation. The final recombinant sEH preparations had no esterase or glutathione *S*-transferase activity which interferes with the CMNPC assay as described below. Human sEH was obtained by expressing cDNA in the baculovirus system in the cell line Spodoptera frugiperda 21. The recombinant protein is an analogue of soluble epoxide hydrolase isolated from human liver in molecular weight, hydrolytic activity, inhibition and immunoreactivity.

### Determination of inhibitory potency (IC_50_) by fluorescent assay (CMNPC assay)^27^

The enzyme (ca. 1 nM human sEH) was incubated at 30 °C with inhibitors ([I]_final_ = 0.4–100,000 nM) for 5 min in 100 mM sodium phosphate buffer (200 μL, pH 7.4) containing 0.1 mg mL^−1^ of BSA and 1% of DMSO. The substrate (cyano(2-methoxynaphthalen-6-yl)methyl *trans*-(3-phenyloxyran-2-yl)methylcarbonate, CMNPC) was then added ([S]_final_ = 5 μM). The activity was assessed by measuring the appearance of the fluorescent 6-methoxynaphthaldehyde product (*λ*_em_ = 330 nm, *λ*_ex_ = 465 nm) at 30 °C during a 10 min incubation (Spectramax M2; Molecular Device, Inc., Sunnyvale, CA). The IC_50_ values, which are the concentrations of inhibitors that reduce activity by 50%, were calculated from at least five different concentrations, each in triplicate, with at least 2 on either side of 50% activity mark.

### Molecular modelling

#### Molecular dynamics simulations

**Figure 3.**
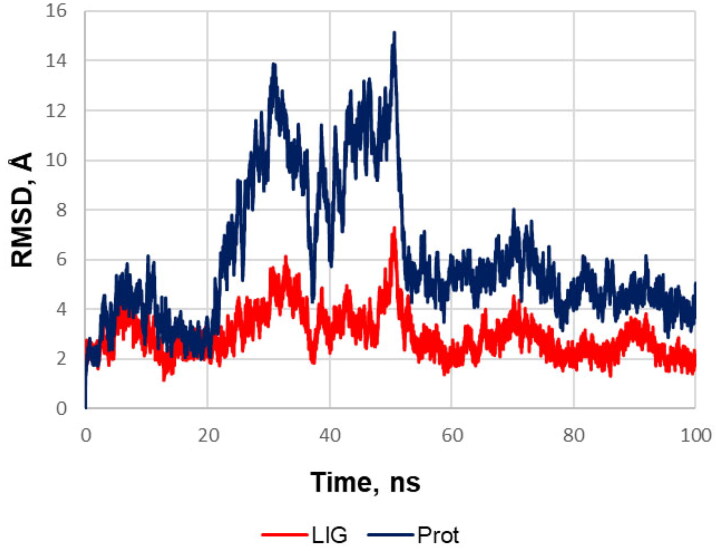
RMSD of the heavy protein atoms during the molecular dynamics simulation of the compound **12** (red) and protein (blue). The plot and the visual analysis of the trajectory indicates that during the simulation of molecular dynamics one monomeric subunit of sEH sharply deviates at the twentieth second and then returns to its original position; while the ligand shifts slightly to a more favourable position in the binding site and from about the fiftieth second the system stability is retained over the course of the production simulation.

A three-dimensional model of the soluble epoxide hydrolase complex with *t*-AUCB (**1a**) (PDB ID: 5AM3)^33^ was obtained from the Protein Data Bank (it was chosen due to a higher resolution compared to similar model of sEH – *t*-AUCB complex: PDB ID: 3WKE^30^) The molecule of *t*-AUCB and substances used for protein X-ray diffraction analysis were removed from the model. The starting structure of the sEH complex with compound **12** was obtained by means of molecular docking (see below). Molecular dynamics simulation was performed in the CHARMM36/CGenFF 4.4 force field^34^^,^^35^ using the GROMACS 2020.3 program.^36^ The initial model of the system was built using the Ligand Reader & Modeller and Solution Builder modules of the CHARMM-GUI web service.^37^ The protein molecule was built into a rectangular periodic box filled with water in the TIP3P model; the distance from the protein to the box boundary was at least 10 Å (approximate total size of the box was 120 × 120 × 120 Å^3^). Randomly selected water molecules were replaced by potassium and chlorine ions to ensure the electrical neutrality of the system and attain the total KCl concentration of 0.15 M. An initial molecular mechanics minimisation (up to 5000 steps) was performed on the central processor, and then the preliminary molecular dynamics equilibration was performed at 300 K temperature for 125 ps at constant volume using a v-rescale thermostat on an NVIDIA GeForce GTX 1080 GPU. Next, a working simulation of molecular dynamics was performed on a GPU at a constant pressure of 1 atm and a temperature of 300 K using a v-rescale thermostat and a Parrinello–Raman barostat (the movements of hydrogens were limited using the LINCS algorithm). The cpptraj software^38^ in the AmberTools 18 package^39^ and UCSF Chimaera 1.15^40^ were used to analyse and visualise the results. Mass-weighted root mean square deviations (RMSD) of the heavy protein atoms during the molecular dynamics simulation of the compound **12** and protein are presented at Figure 3.

**Figure:**
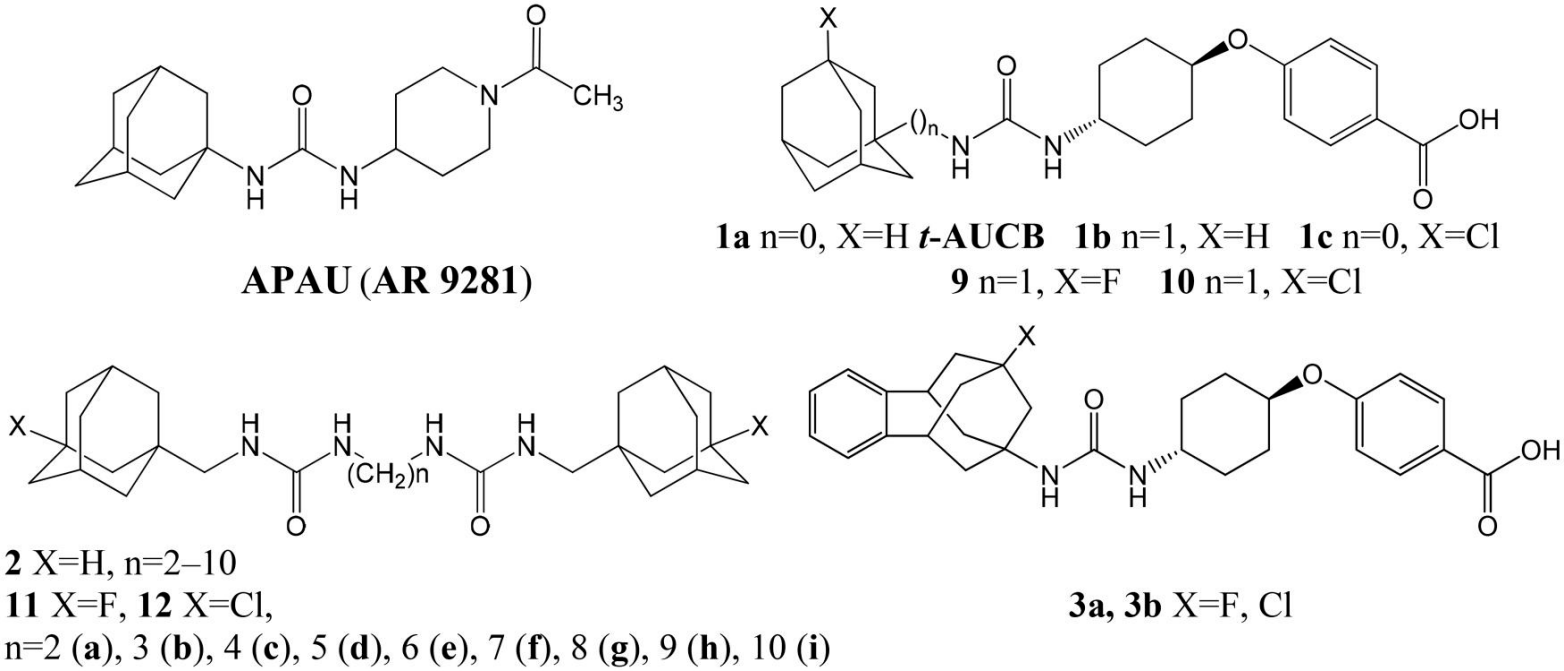


#### Molecular docking

Molecular docking of the compounds was carried out in a 3D model of the sEH – *t*-AUCB (**1a**) complex (PDB: 5AM3) (substances used for protein X-ray diffraction analysis and *t*-AUCB molecule were preliminarily removed from the model). The protein atoms were charged according to the standard Kollman method using the AutoDock Tools 1.5.6 program. The 2D structures of the ligands were transformed into 3D ones, and the geometry was optimised by molecular mechanics in the Amber ff14SB force field using the Gasteiger charge model in the USCF Chimera 1.15 program.^40^ The docking procedure was carried out using the AutoDock Vina 1.1.2 program^41^ (grid box size: 12.75 Å × 15.0 Å × 21.75 Å, coordinates of the centre: x = 16.111 Å, y = 9.722 Å, z = 13.582 Å, exhaustiveness: = 20, energy range: = 4). The ligand – protein complexes with the best values of the scoring functions were selected. The structures of the complexes were visualised using the UCSF Chimaera 1.15 program.^40^

2D interaction plots were obtained using Discovery studio visualiser (BIOVIA, San Diego, CA, USA).^42^
